# Gastrointestinal Diets with Different Fiber Compositions and Energy Densities for Management of Chronic Inflammatory Enteropathy in Dogs: A Prospective, Multicenter Study

**DOI:** 10.3390/ani16203163

**Published:** 2026-10-09

**Authors:** Julie Rochon, Mariacristina Vecchio, Danielle Dito, Nilüfer Oruz, Hélène Charles

**Affiliations:** Royal Canin Research & Development Centre, 30470 Aimargues, France

**Keywords:** chronic enteropathy, gastrointestinal signs, vomiting, diarrhea, colitis, inflammatory bowel disease, gastrointestinal diet, highly digestible diet, fiber

## Abstract

Dogs with chronic gastrointestinal (GI) signs are commonly presented to veterinarians. This Italian study investigated the effectiveness of three dry, highly digestible veterinary GI diets fed for 60 days to control chronic inflammatory enteropathy (CIE). Adult dogs with a Canine Chronic Enteropathy Clinical Activity Index score ≥ 5, modified to exclude albumin concentration (mCCECAI), were assigned a GI high-fiber diet (*n* = 21) if GI signs were primarily large bowel, a GI moderate-calorie diet (*n* = 22) if signs were small or mixed small/large bowel and body condition score (BCS) was > 5, or a GI diet (*n* = 18) if signs were small or mixed small/large bowel and BCS was ≤ 5. Least-squares mean mCCECAI scores and owner-reported GI signs improved significantly in all diet groups between baseline and Day 30 (73.5–79.8% and 72.1–81.7%, respectively, *p* < 0.0001) and baseline and Day 60 (85.0–86.3% and 89.8–93.0%, respectively, *p* ≤ 0.0001). Body weight and BCS were stable, except for an 8.0% BCS increase in the GI diet group. There were no adverse events considered likely to be diet-related. A similar Australian study of the high-fiber GI diet had consistent results. These data suggest that each diet is equally effective for its indicated use, inducing rapid and sustained improvements in CIE signs.

## Simple Summary

Chronic gastrointestinal (GI) disorders are common in dogs and often managed with therapeutic diets. The Italian study reported here investigated the effectiveness of three dry, highly digestible diets fed for 60 days to dogs with chronic signs of a GI disorder: GI high-fiber diet for 21 dogs with primarily large bowel signs, GI moderate-calorie diet for 22 overweight dogs with small or mixed small and large bowel signs, and a GI diet with a higher calorie content for 18 underweight dogs with small, or mixed small and large bowel signs. There were similar significant reductions in clinical signs in all diet groups on Day 30 that were sustained to Day 60. Mean improvements ranged from 73.5% to 85.0% as assessed by veterinarians, and from 72.1% to 93.0% as assessed by dog owners. There were no significant changes in average body weight or body condition score (BCS) at Day 60, except for an 8.0% increase in average BCS in the GI diet group. Results from a similar Australian study that investigated only the GI high-fiber diet were consistent with the Italian findings. In conclusion, three GI diets with distinct compositions successfully targeted dogs with different constellations of chronic GI signs.

## 1. Introduction

Gastrointestinal (GI) signs are a common reason for dogs to be presented to primary veterinary practices. In the Dog Aging Project in the USA (N = 43,517), noninfectious, clinical-sign-based GI disorders were reported to have occurred at any time in 5% of dogs and inflammatory GI disorders in 1% of dogs [1]. In primary veterinary practices in the UK, at the most precise diagnostic level, diarrhea and vomiting have been ranked the 6th and 7th most common disorders, respectively, and at a grouped-diagnostic level, enteropathy accounted for approximately 10% of diagnoses [2]. Some of these dogs will meet the diagnostic criteria for chronic inflammatory enteropathy (CIE), which is a group of GI disorders characterized by persistent or recurrent signs of GI disease, such as diarrhea, vomiting, reduced appetite, and weight loss; evidence of mucosal inflammation; and absence of specific etiologies, such as infections, parasitic infestations, anatomical disorders, or neoplasia [3,4,5,6]. Categorization of CIE is based on response to treatment, and accordingly CIE is often described as food-responsive, immunosuppressant-responsive, or non-responsive [3,4].

Food-responsive CIE is the most prevalent type of CIE [3,4,6,7,8], and a GI diet is the first treatment recommended in the diagnostic algorithm [3]. GI diets are formulated to modulate the macro- and micronutrient composition of the diet qualitatively or quantitatively, targeting different pathological mechanisms of GI signs. First-choice, mainstream (standard) veterinary therapeutic GI diets that promote stool quality and normal stool frequency are primarily highly digestible diets with bioavailable, intact proteins from common sources, highly digestible starch, and increased concentrations of sodium and potassium. Variations include a reduced fat concentration, and enhancement of the type and balance of insoluble and soluble fiber content [9,10]. Veterinary therapeutic diets with hydrolyzed protein or selected protein diets are also widely used in GI conditions, primarily addressing GI signs associated with adverse food reactions or any conditions in which low antigenicity of the diet could be useful [3,9].

Most of the dietary research studies in dogs with GI signs have investigated elimination diets [6,7,11,12,13] or hydrolyzed protein diets [7,13,14,15,16,17,18,19,20,21] formulated for CIE. There is a need for more evidence on the effectiveness of conventional veterinary GI diets that might be more appropriate as a first diet switch for dogs with signs of CIE.

The aim of the current study was to investigate the effectiveness of three dry veterinary GI diets formulated for the control of non-specific CIE in dogs. The diets varied in fiber content and energy density to target dogs according to their predominant clinical presentations. The data were collected in two prospective, interventional, non-randomized, multicenter field studies in Italy and Australia, in which dogs were fed one of the diets as their sole food source for 60 days. Clinical effectiveness was evaluated primarily by improvements in a modified Canine Chronic Enteropathy Clinical Activity Index (mCCECAI), body weight and body condition score (BCS). Owners’ observations of clinical signs, food consumption, and quality of life were also collected.

## 2. Materials and Methods

### 2.1. Study Design

The study protocols were approved by Royal Canin’s Ethics Committee, and the Australian protocol was approved by the Animal Ethics Committee of Invetus, the contract research organization. Studies were conducted in compliance with national and local regulations where applicable. All scheduled procedures for the study were standard clinical assessments, except possibly for blood sampling to measure albumin concentration, although their frequency may have been higher than that for dogs with similar clinical presentations outside of a clinical trial. Informed consent for participation was obtained from all owners of dogs enrolled in the study. No owner-identifying information is presented.

The Italian study was a prospective, non-randomized, multi-centered study to investigate the effectiveness of three dry, commercially available, veterinary GI diets (Section 2.3). Additional data were derived from a prospective, multicentered, single-armed Australian study that followed the same protocol as the Italian study, except where noted below. The diets were fed for 60 days (±3 days); there were scheduled clinic visits on Day 0 (baseline), Day 30 (±3 days), and Day 60 (±3 days), and a telephone consultation on Day 15 (±3 days). The Italian study was conducted between 21 December 2022 and 28 June 2023, and the Australian study between 26 November 2022 and 31 July 2023. All dogs were client-owned and lived with their owners for the duration of the study.

### 2.2. Animals: Inclusion and Exclusion Criteria

Client-owned, adult (age > 18 months for giant breeds and > 12 months for other breeds) male and female dogs of any breed weighing > 1 kg with CIE could be considered for study inclusion by the treating veterinarian. A diagnosis of CIE required either (1) at least two episodes per week for 2 weeks or more of soft or liquid feces and at least one of the following concomitant signs: increased frequency of defecation, increased fecal volume, or vomiting, or (2) a history consistent with CIE, i.e., at least one veterinary clinic visit for the same reason or same GI signs within the past year (excluding GI episodes known to be caused by the consumption of inappropriate substances, stress, or concomitant disease). The presence of other possible GI signs, specifically mucus or blood in the stools (melena, or hematochezia, or both), tenesmus, unwanted weight loss over the last month, and loss of appetite, was also recorded. GI signs could be intermittent or continuous (daily occurrences). Only dogs with a modified CCECAI (mCCECAI) score ≥ 5 on Day 0 (mild-to-severe CIE) were included. The mCCECAI was adapted from the CCECAI (Appendix A Table A1, maximum total score = 24) [6] by excluding the serum albumin component, as not all veterinarians had blood test results at the time of enrollment. Therefore, for consistency, mCCECAI was used throughout the study.

Dogs could not be enrolled if they had received systemic antibiotic therapy within the previous 30 days, long-acting immunosuppressive drugs within the previous 6 weeks, or any systemic immunosuppressive drugs within 21 days before screening. Animals with low or suspected low serum cobalamin concentrations could be supplemented with vitamin B12 unless the deficiency was suspected or confirmed to be hereditary selective cobalamin malabsorption. Other clinical veterinary dietary interventions had to be discontinued the day prior to enrolment, e.g., probiotics and psyllium. Other non-inclusion criteria included hospitalization for GI signs, concurrent disease, or GI signs caused by another disease at baseline, e.g., chronic kidney disease, exocrine pancreatic insufficiency, acute pancreatitis, infectious disease and endoparasites, and hyperthyroidism. Exclusion of other diseases at baseline was based on the treating veterinarian’s overall assessment of clinical signs and blood test results. There was no limitation on additional or repeat blood tests at any time during the study; these were at the discretion of each veterinarian.

If a dog was not receiving regular anthelmintics, it could not be enrolled until 3 days after completion of veterinary-prescribed anthelmintic treatment, which included treatment for *Giardia* and *Trichomonas* if these were possible causes of the GI signs.

Dogs being fed hydrolyzed protein diets or other hypoallergenic diets, including homemade diets, had to be able to switch to a standard GI diet, and owners had to be willing to discontinue the practice of feeding treats, snacks, and supplements intended for GI disease or other conditions.

Animals were removed from the study if they had anorexia or reduced appetite for three consecutive days with consumption of <80% of the daily ration. Concurrent medications during the study were only permitted if they were required as part of standard management practice. In addition, use of such medications was reviewed by a qualified veterinarian (the Royal Canin Pet and Product Scientific Specialist) to assess their potential effects on clinical GI signs; if an impact on study outcomes was plausible, the dog was removed from the analysis.

### 2.3. Diets and Feeding

In the Italian study, dogs were assigned to one of the study diets (Table 1) by the treating veterinarian according to their clinical presentation. Royal Canin^®^ Gastrointestinal High Fibre diet was indicated for dogs with predominantly or solely large bowel signs, which typically include increased frequency of defecation, normal to reduced fecal volume, mucus, hematochezia, and tenesmus [6]. Royal Canin^®^ Gastrointestinal Moderate Calorie diet was indicated for dogs with small or mixed small and large bowel signs and a BCS > 5, or dogs with a pancreatic-specific lipase concentration indicative of chronic pancreatitis (Appendix A Figure A1) [22]. Royal Canin^®^ Gastrointestinal diet was indicated for dogs with small or mixed small and large bowel signs and a BCS ≤ 5. Signs characteristic of small bowel disturbances include increased fecal volume, normal fecal frequency, melena, and weight loss [6]. In Australia, only dogs eligible for the Royal Canin^®^ Gastrointestinal High Fibre diet could be enrolled because the other diets were not then available in Australia.

The daily food ration was based on Day 0 body weight and BCS according to the manufacturer’s feeding guide. The food ration could be provided as a single meal or multiple meals or be accessible at all times. Dogs were transitioned gradually to the study diet over the first 4 days, after which only the study diet was fed. On Day 30 (±3), the diet could be switched if there was a protocol-defined partial or non-response to the current treatment diet (Section 2.4).

### 2.4. Study Assessments

Treating veterinarians ascertained study eligibility based on a combination of the dog’s history, a physical examination on Day 0, and the results of a 12-h fasted blood sample taken on Day 0 for a complete blood cell count and analysis of standard biochemistry analytes plus canine pancreas-specific lipase (Spec cPL), trypsin-like immunoreactivity (TLI), vitamin B12, and vitamin B9 (folate) (BiEsse A-Laboratorio di Analisi Veterinarie SRL, Milano, Italy; Idexx Laboratories, Brisbane and Sydney, Australia; Vetnostics, North Ryde, Australia). Physical examinations were repeated on Days 30 and 60.

The primary study endpoints were changes from baseline to Day 30 and Day 60 in the mCCECAI score, owner-reported clinical score (Italian study only), body weight, and BCS (9-point scale ranging from 1 = too thin to 9 = obese [Appendix A Figure A1] [22]). Owner-reported clinical scores were also ascertained in the telephone consultations. These clinical scores were the sum of the owner’s ratings for their dog’s attitude, appetite, vomiting, stool consistency, and stool frequency on a scale from 0 (normal) to 3 (severely different from normal) (maximum total score = 15; Appendix A Table A2).

Secondary endpoints included response to treatment at Day 30. In both studies, dogs were classified by the treating veterinarian as complete responders if clinical signs had completely abated and defecation was assessed as normal by the owner, and/or if the mCCECAI score was <3. A dog was considered a partial responder if the frequency and severity of clinical signs had decreased by at least one-third (but, in the owner’s opinion, the pattern of defecation had not completely returned to normal) and/or when the mCCECAI score was between 3 and 5. A non-responder was a dog that had no improvement in clinical signs, or only marginal improvement (less than one-third reduction) and/or a mCCECAI score > 5. The veterinarian could choose to withdraw a partial responder from the study at Day 30, in which case the dog would be classified as a non-responder. The protocol recommended that non-responders be withdrawn from the study.

Food consumption was reported by owners during the telephone consultation and on Days 30 and 60 as the amount of the daily ration being eaten. In the Italian study, this was rated on a scale of 1 to 9, where 9 indicated that the entire ration was being consumed. In the Australian study, owners selected from response options of 0%, 25%, 50%, 75%, and 100% consumption of the daily ration.

Owners evaluated their dog’s quality of life on a scale from 0 (poor quality) to 10 (excellent quality). They were also asked to rate the discomfort caused by GI problems on a scale from 0 (no discomfort) to 10 (extreme discomfort). Responses to these questions were requested at Day 0 in the Italian study and at Days 0, 15, 30, and 60 in the Australian study.

Adverse events (AEs) were monitored and recorded at all visits and consultations (scheduled or unscheduled). Owners were advised to call the clinic at any time during the study if their dog’s clinical signs were of concern so that an additional visit could be arranged. Veterinarians reported AEs, including weight loss or gain, or other changes of concern resulting in an additional clinic visit. The veterinarian used clinical judgement to determine whether the AE was related to the study diet.

### 2.5. Statistical Analysis

A power calculation was conducted using G*Power 3.1.9.2 software to determine the target sample size. Sample size was based on the power to detect a significant difference between diet groups in the number of dogs that achieved ≥75% reduction in mCCECAI at Day 60 compared with Day 0 and/or a score < 3 at Day 60. The calculation assumed an effect size using Cohen’s $d=\frac{\mu_{1}-\mu_{2}}{\sigma }=1.0$, a significance level α=0.05, and a statistical power of 1−β=90%, utilizing a one-sample, two-sided t-test. The calculated sample size was 13 dogs; this was increased by 20% to a target enrollment of 16 dogs per diet to allow for potential dropouts or removals.

The Italian data for all three diet groups were analyzed separately from the Australian data. Where endpoints were the same in the Italian and Australian studies, there was a separate additional analysis for a pooled Italian and Australian dataset for the GI high-fiber diet. Where endpoints were similar but not exact enough to be combined, the Australian data were analyzed as a discrete group. Statistical Analysis System (SAS), Version 9.4, was used for all the statistical analyses.

Data from enrolled animals were included in the analysis up to Day 60 or up to the time of early discontinuation. Each dog represented an experimental unit. Repeated-measures linear mixed models tested main effect hypotheses at a two-sided 0.05 level of significance, and interactions between effects were tested at a two-sided 0.1 level of significance. For each primary endpoint (mCCECAI score, owner-adapted clinical score, body weight and BCS) and the continuous food consumption endpoint, the fixed effects were Day and Diet or Day and Country (pooled Italian + Australian data) and their interaction. The repeated factor for within-subject correlation was Day (nested within study center). Covariance structures were considered (TOEP, CSH, ARH(1), TOEPH, UNR, ARMA(1,1), and UN), and the model with the lowest Akaike Information Criterion (AIC) value was selected. Residuals distributions were checked and indicated that mathematical transformations were not required. Arithmetic least squares means (LSMs derived from the repeated-measures linear mixed models) with 95% confidence intervals (CIs) were calculated for each day and diet of the aforementioned endpoints together with LSM changes from Day 0. No formal adjustment for multiple comparison was applied.

For each secondary endpoint and diet, a one-sided binomial exact test was performed using a 5% significance level, with the null hypothesis assuming equal probabilities for each response category, to test whether there was a significant majority in any response category compared to the others. Categorical responses were analyzed by logistic regression with a cumulative logit link function with a fixed effect for the treatment diet. The odds ratio (OR) (95% CIs) between treatment diets of higher scores versus lower scores in the categorical responses are presented.

## 3. Results

### 3.1. Study Populations

In the Italian study, a total of 61 dogs were recruited across ten study centers, one of which enrolled seven dogs, and the other nine enrolled six dogs each. There were 21 dogs assigned to the GI high-fiber diet, 22 dogs assigned to the GI moderate-calorie diet and 18 dogs assigned to the GI diet (Table 2). Across all Italian diet groups, ages ranged from 1.0 to 14.0 years (median 6.0 years), body weights ranged from 2.4 to 54.0 kg (median 20.2 kg), and BCS ranged from 1.0 to 9.0 (median 5.0). In the moderate-calorie diet group, four dogs had not been assigned per protocol as they each had a BCS < 6. There were 41 breeds, with 35 (57.4%) females and 26 (42.6%) males in the study. Before commencement of the study, dogs were being fed a regular maintenance diet (*n* = 42 dogs), a mono-protein diet (*n* = 5), a weight-management diet (*n* = 4), a GI (digestive care) diet (*n* = 2), a GI low-fat diet (*n* = 2), a hydrolyzed-protein diet (*n* = 2), a sensitivity diet for food intolerances (*n* = 1), a urinary health diet (*n* = 1), a mobility diet (*n* = 1), or a home-prepared diet (*n* = 1). Other than antiparasitic medications, none of the dogs in the Italian study were treated with concomitant medications.

All Italian dogs completed the study, except for one dog in the GI diet group that discontinued on Day 31 because of food refusal. None of the dogs switched their diet during the study. Data from all enrolled dogs were included in the analysis.

In the Australian study, there were four study centers, two of which enrolled two dogs each, one enrolled three dogs, and one enrolled seven dogs. Australian dogs had a median age of 2.9 years (range, 1.3–12.5 years), with body weights ranging from 4.2 to 36.8 kg (median 17.7 kg) and BCS ranging from 2.0 to 7.0 (median 4.8). One Australian dog was enrolled with an mCCECAI score of 3 on Day 0, but a review of the baseline case report forms concluded that this dog was likely to have CIE. Enrolled dogs represented 11 breeds, with 8 (57.1%) females and 6 (42.9%) males (Table 2). No data was collected on previous diets. Nine dogs in the Australian study received medications other than antiparasitic treatments. Three dogs had ongoing treatments: one dog with thyroxine, omeprazole, gabapentin, and a *Biota orientalis* joint supplement; another dog with fluoxetine, omeprazole, gabapentin, maropitant (as required), and propentofylline; and the third dog with fluoxetine. Single doses or short treatment courses for other dogs were clonidine (*n* = 1), diazepam (*n* = 1), gabapentin (*n* = 3), cetirizine (*n* = 1), tramadol (*n* = 1), trazodone (*n* = 1), maropitant (*n* = 1), ondansetron (*n* = 1), and a probiotic and postbiotic treatment (*n* = 1).

All 14 Australian dogs were fed the GI high-fiber diet, and of these, four discontinued the study: the first on Day 15 due to allergy-like signs; one on Day 16 due to a palatability problem, an atopic reaction and anxiety; one on Day 37 due to GI disease that was not compatible with study continuation and/or required treatment incompatible with the study, and a palatability problem; and the last on Day 42 due to inappetence and weight loss. Data from all enrolled Australian dogs were included in the analysis.

### 3.2. Modified CCECAI (Vet-Reported Clinical Scores)

The mCCECAI scores decreased over time relative to the baseline in all treatment groups (Table 3 and Appendix A Figure A2). The LSM changes from baseline in mCCECAI score were all statistically significant at all timepoints for all diet groups (*p* < 0.0001) (Table 3). Improvements at Day 30 and Day 60 were 73.5% and 85.9%, respectively, in the Italian GI high-fiber group; 79.4% and 86.3%, respectively, in the GI moderate-calorie group; 79.8% and 85.0%, respectively, in the GI group; and 69.6% and 70.8%, respectively, in the pooled Italian + Australian GI high-fiber group. The improvements in mCCECAI in the Australian GI high-fiber group were 64.7% (*p* < 0.0001) and 53.4% (*p* = 0.0001) at Day 30 and Day 60, respectively.

### 3.3. Owner-Reported Clinical Scores

Owner-reported clinical scores were recorded only in the Italian study, and for these dogs, the mean scores declined over time (Table 4 and Appendix A Figure A2). The LSM changes from baseline at all timepoints were statistically significant (*p* < 0.0001). Improvements at Day 30 and Day 60 were 72.1% and 89.8%, respectively, in the GI high-fiber group; 81.7% and 93.0%, respectively, in the GI moderate-calorie group; and 79.8% and 90.2%, respectively, in the GI group.

### 3.4. Body Weight

The mean body weight of each diet group remained constant throughout the Italian study. The LSM changes in weight from baseline at Day 30 and Day 60 were 0.2% (*p* = 0.633) and −0.2% (*p* = 0.772), respectively, in the GI high-fiber group; 0.1% (*p* = 0.943) and −0.5% (*p* = 0.6713), respectively, in the GI moderate-calorie group; and −0.2% (*p* = 0.812) and −0.6% (*p* = 0.593), respectively, in the GI group (Appendix A Table A3). In the pooled Italian + Australian GI high-fiber group, the LSM change in weight from baseline was −0.7% at Day 30 (*p* = 0.283) and −0.8% at Day 60 (*p* = 0.214). The Australian GI high-fiber group also had no significant changes in weight, with the LSM change from baseline being −2.2% (*p* = 0.156) at Day 30 and −1.9% (*p* = 0.237) at Day 60.

### 3.5. Body Condition Score

There were no significant changes in the LSM BCS for Italian dogs fed the GI high-fiber diet or the moderate-calorie diet, although there was an increase in the pooled Italian + Australian group at Day 30. Changes from baseline in LSM BCS at Day 30 and Day 60 were 1.9% (*p* = 0.329) and 0.9% (*p* = 0.720), respectively, for the Italian GI high-fiber group; −1.5% (*p* = 0.340) and −2.3% (*p* = 0.295), respectively, for the Italian GI moderate-calorie group; and 7.3% (*p* = 0.011) and 6.0 (*p* = 0.151), respectively, for the Italian + Australian GI high-fiber group (Appendix A Table A4). In the GI diet group, the LSM BCS was unchanged at Day 30 compared with baseline (3.0% increase [*p* = 0.292]) but increased significantly by 8.0% at Day 60 (*p* = 0.046). There were no significant changes in BCS in the Australian high-fiber group, with the LSM change from baseline being 12.3% (*p* = 0.123) at Day 30 and 9.3% (*p* = 0.376) at Day 60.

### 3.6. Food Consumption

In the Italian study, more than 60% of dogs in all groups at all timepoints from Day 15 had consumption scores of 9 (all food consumed), and scores for all dogs were ≥7, with the exception of a score of 1 at Day 30 for one dog in the GI group, which consequently discontinued at Day 31 (Appendix A Table A5). The ORs (95% CI) for higher consumption scores at Days 15, 30, and 60, were 0.3 (0.1–1.3), 0.9 (0.2–4.0), and 1.1 (0.3–4.0), respectively, for the GI high-fiber diet versus the GI moderate-calorie diet; 0.6 (0.1–3.0), 0.5 (0.1–2.1), and 1.9 (0.4–7.9), respectively, for the GI high-fiber diet versus the GI diet; and 2.1 (0.51–8.53), 0.5 (0.13–2.09), and 1.7 (0.4–7.0) for the GI moderate-calorie diet versus the GI diet. None of these ORs were statistically significant.

In the Australian study, owners reported food consumption as a percentage of the daily study ration, selecting from one of six options. At Day 30 (*n* = 12), food consumption was estimated to be 100% in eight dogs, 80–95% in two dogs, and 50% in one dog and not reported in one dog. At Day 60 (*n* = 10), food consumption was reported to be 100% in seven dogs, 80–95% in two dogs, and 75% in one dog.

### 3.7. Response to Diet

In the Italian study, complete responses were reported at Day 30 in 38.1%, 63.6%, and 61.1% of dogs receiving the GI high-fiber diet, GI moderate-calorie diet, and GI diet, respectively (Table 5). The remaining dogs had partial responses, and there were no non-responders. There were no statistically significant differences between diets for the odds of being a complete responder (Table 5). Within diet groups, there were no statistically significant differences in the probability of being a complete responder versus a partial or non-responder (*p* = 0.138, *p* = 0.101, and *p* = 01173 for the GI high-fiber, GI moderate-calorie, and GI diet, respectively).

Diet responses in the Australian study were required only at Day 60 in the case report forms. Of the ten dogs with Day 60 visits, seven were complete responders, and the other three did not have this outcome recorded. Of the four dogs that discontinued early, the response at the time of discontinuation was not recorded for two dogs, was partial for one dog, and was complete for another.

### 3.8. Quality of Life

In the Italian study, discomfort caused by GI problems and quality of life were evaluated only at Day 0; mean scores for each of these were similar across diet groups (Table 2). In the Australian study, the mean discomfort score decreased significantly from baseline at all timepoints: −52.1% (*p* = 0.0171) at Day 14, −66.1% (*p* = 0.0009) at Day 30, and −77.4% (*p* < 0.0001) at Day 60 (Appendix A Table A6). By Day 60, the mean discomfort was minimal, with an LSM (95% CI) of 1.2 (0.5–1.9). Quality of life scores in the Australian study did not change significantly from the baseline at any timepoint (Appendix A Table A7).

### 3.9. Adverse Events

No serious AEs were reported in any of the study dogs. In the Italian study, one dog in the GI diet group had an AE of soft feces and increase in stool frequency, which self-resolved in 2 days. Four dogs were withdrawn from the Australian study after AEs, which were considered at most possibly related to the study diet. These AEs were food refusal (*n* = 1); diarrhea, vomiting, inappetence, and water refusal (*n* = 1); inappetence, pruritus with moderate erythema and hyperkeratosis, and anxiety (*n* = 1); and watery eyes, reverse sneezing, and decreased appetite (*n* = 1). Two other Australian dogs had episodes of vomiting (unknown or unlikely to be related to the study diet) that spontaneously resolved, and one dog had weight loss possibly related to diet that was treated with an increase in the food ration.

## 4. Discussion

In this prospective two-study field trial, three GI diets were effective in significantly improving the clinical signs of CIE after 30 days of feeding, which was sustained until Day 60 with continued feeding. Improvements were observed in both the mCCECAI scored by the veterinarian and the owner-reported clinical scores that evaluated the dog’s attitude, appetite, vomiting, stool consistency, and stool frequency. In the Italian study, the improvements were ≥75% in the mCCECAI and owner-reported clinical scores compared to baseline in the GI high-fiber group on Day 60, and in the GI moderate-calorie and GI groups on Days 30 and 60. The mCCECAI score improved by approximately 70% in the pooled Italian + Australian GI high-fiber group. In the Italian study, which had protocol definitions for overall response, all dogs responded to treatment, and there was a complete response in >60% of dogs fed the GI moderate-calorie diet or the GI diet. Diets were assigned to different dogs depending on their clinical presentation, and in pairwise comparisons between groups, the diets were similarly effective.

The study was not randomized, which may introduce selection bias and therefore represent a limitation. However, the primary objective of the study was not to compare the three diets, but to evaluate the effectiveness of each diet when used according to their intended recommendations. Dogs were therefore allocated by the treating veterinarian based on predefined clinical criteria, including the predominant localization of gastrointestinal signs and body condition score, an approach that reflects real-world veterinary clinical practice. Similarly, while clinical scoring systems provide a standardized method to quantify disease severity and monitor treatment response, they can be susceptible to observers’ bias. The owner-reported clinical score was included to capture observations made in the home environment, where dogs spend most of their time. To reduce the risk of bias, scoring guidance was provided to the owners, and clinical outcomes were also assessed by veterinarians using the modified CCECAI score, based on a validated clinical scoring system.

The GI high-fiber diet was formulated primarily for disturbances in the functioning of the large intestine, where bacterial fermentation of fiber and most of the water absorption from digesta occurs [24]. The physiological advantages of dietary fiber translate into systemic clinical benefits. These include modulation of immune responses, synthesis of vitamins, anti-inflammatory effects, and enhanced insulin sensitivity. Additionally, fiber optimizes the absorptive capacity of colonocytes, facilitates fecal bulking, and reduces the accumulation of toxic metabolic byproducts. Dietary fibers differ in solubility and fermentability, and thereby differentially affect the physical and chemical environment of the GI tract and the composition and diversity of the gut microbiota [25]. Relatively insoluble fiber with low fermentability, e.g., cellulose, adds organic bulk to stools without excess water, in contrast to soluble fiber with moderate fermentability, e.g., psyllium, which retains moisture in a gel matrix, thereby increasing and softening fecal bulk [24,25,26]. Undigested fermentable fiber from the upper GI tract is a substrate for microbes in the large intestine, and among the metabolites, short-chain fatty acids (SCFAs) are particularly beneficial to the host. SCFAs provide rapidly absorbed energy sources for enterocytes, enhance the mucosal barrier, modulate regulatory T-lymphocytes to protect against mucosal inflammation, and inhibit some pathogenic bacteria by reducing GI pH [4,24,25,26]. While fiber inclusion offers significant therapeutic benefits, clinicians must consider potential physiological drawbacks, such as increased satiety, reduced overall nutrient digestibility, and delayed gastric emptying due to high viscosity. Consequently, customizing the total dietary fiber load, selecting specific fiber classes, and balancing the soluble-to-insoluble ratio are critical steps to serve the individual needs of each dog [27]. A healthy balance of fiber is particularly important for dogs with GI signs because a relative decrease in the abundance of SCFA-producing microbes and the presence of dysbiosis are characteristic of dogs with CIE or inflammatory bowel disease [28,29,30]. It is often not possible to attribute improvements in GI signs to a particular dietary component, and the benefits of GI diets depend on the nature and balance of functional nutrients. Nevertheless, several studies have demonstrated the benefits of high-fiber diets or selective fiber supplementation in healthy adult dogs [4,31,32,33] for the treatment of large-bowel diarrhea [34,35,36,37].

The study diets were supplemented with fructo-oligosaccharides (FOS) and a brewer’s yeast source of mannan-oligosaccharides (MOS). These are fructan and mannan fibers that act as prebiotics by virtue of their selective utilization by microbiota to confer health benefits to the host [10]. FOS and MOS selectively promote the growth of beneficial SCFA-producing bacteria in the GI tract, e.g., *Lactobacillus* and *Bifidobacterium* species [38], and in dogs, dietary supplementation with FOS and MOS has positive effects on microbial and immunological indices of gut health [39].

All diets used in this study were highly digestible GI diets. A highly digestible diet is characterized by a digestibility of ≥85%, 87%, and 90% for proteins, lipids, and carbohydrates, respectively [27,40]. This characteristic of the study diets was intended to promote healthy functioning of both the small and large intestines, including effective digestion and absorption of nutrients, reduction in the antigenic and inflammatory potential of proteins, and avoidance of osmotic diarrhea and oversupply of fermentation substrates to the large intestine [24,26]. Proteins escaping digestion in the small intestine can be fermented to deleterious metabolites in the large intestine, including branched-chain fatty acids [41,42]. The high digestibility of therapeutic diets for the treatment of CIE can be achieved in part by the hydrolysis of the protein content, and hydrolyzed protein diets are often effective for food-responsive CIE [7,13,14,15,16,17,18,19,20,21,43]. The study diets contained some hydrolyzed protein, but not all proteins were hydrolyzed because the target population was dogs with non-specific CIE. The digestibility of the GI high-fiber diet was less than that of the other two diets as would be expected [44], not only because fiber is by definition indigestible by the host, but also because it can increase transit time and affect the digestibility of dietary protein and fat [45,46].

Formulating the fat content of GI diets requires consideration of the likely tolerance of an individual to fat, the positive effects of animal fat on palatability, and the high energy content of fat. The GI diets in this study were not designed to be low-fat, and dogs with conditions that would require a low-fat diet were excluded. The GI diet had the highest fat content and energy density, which were intended to reduce meal volume and intestinal load. This diet was assigned to dogs with BCS at or below ideal, and the statistically significant increase in BCS during the study supported this approach. The GI moderate-calorie diet had the lowest fat content, which helped achieve a lower energy density that would facilitate weight management in dogs with a body condition greater than the ideal. However, in the context of the study, this diet was fed to maintain body weight because the metabolic effects of weight loss could confound the interpretation of clinical signs. Therefore, stable body weight and BCS in the GI moderate-calorie diet was intentional. The study diets contained rich sources of the omega-3 fatty acids eicosapentaenoic acid (EPA) and docosahexaenoic acid (DHA), included for their well-established benefits in the production of anti-inflammatory modulators and inflammation-resolving mediators [47]. Dietary fish oil and microalgae oil (*Schizochytrium* sp.) both contain EPA and DHA. Microalgae oil is considered a sustainable source [45] that is palatable and can increase the digestibility of a diet [48].

This field research was designed to investigate the effectiveness of commercially available diets on GI signs in dogs managed in their home environments. The study design balanced strict eligibility criteria to avoid potentially confounding effects of other health conditions and concomitant medications, with the clinical judgement of each veterinarian for their individual cases. Although it might be seen as a limitation that four dogs with BCS < 6 were fed the GI moderate-calorie diet rather than the GI diet, this reflected the veterinarian’s assessment of the most appropriate diet for these individuals at the time. None of the veterinarians decided to switch diets, although this was permitted in the study protocol. The formulations were based on nutrients with known functional benefits; however, in the absence of control diets, the effects in this study cannot be directly attributed to specific dietary components distinct from the overall formulation. The diets are indicated for CIE, and at least three trials with different diets are recommended before concluding that the CIE is not food-responsive [3]. It is possible that partial responders in this study would have responded completely if switched to one of the alternative study diets or to a hydrolyzed protein diet. The mCCECAI was used as a primary endpoint because CCECAI is a validated [6] and guideline-recommended, semi-objective measure of the severity of GI disease [3]. The owner-reported clinical score was not a validated tool but provided insights from owners who would be observing their dogs daily. The definitions of complete and partial responses did not match those of remission or partial remission of CIE, respectively, and were intended to reflect the owner’s observations of clinical signs. Slight differences in the protocols for the Italian and Australian studies limited the scope for combined analysis; however, the pooled GI high-fiber group corroborated the results of the Italian-only GI high-fiber group.

In summary, these studies show the effectiveness of three GI diets in the treatment of non-specific CIE. Improvements in clinical signs were rapid and sustained. The data suggests that each diet is equally appropriate for dogs with CIE when the diet choice for an individual is based on indicated use and clinical judgement, which includes the interpretation of GI clinical signs as large intestinal or mixed small and large intestinal, and BCS.

## 5. Conclusions

Three gastrointestinal diets with distinct compositions—differing in fiber content and energy density—were effective in managing dogs with a variety of chronic gastrointestinal signs, resulting in rapid and sustained clinical improvement. This suggests that each diet is equally suitable for dogs with chronic inflammatory enteropathy (CIE), supporting a pragmatic approach to initial diet selection when initiating dietary treatment trials, with a choice based on clinical signs (interpreting GI symptoms as indicators of small bowel, large bowel, or mixed disease) and patient history. Overall, the clinical benefits observed with these three gastrointestinal diets are essential to the management of CIE and confirm their important role in the treatment plan for dogs with this condition.

## Figures and Tables

**Table 1:** Composition of study diets.

	GI High-Fiber Diet		GI Moderate-Calorie Diet		GI Diet	
Analytical Constituents	% as Fed	g/Mcal	% as Fed	g/Mcal	% as Fed	g/Mcal
Moisture	9.5	-	9.5	-	8.0	-
Protein	23.2	73.2	23.0	63.7	25.0	60.6
Fat	16.1	50.8	11.0	30.5	20.0	48.5
NFE	32.7	103.1	47.8	132.3	35.0	93.8
Crude fiber	11.1	35	2.3	6.4	1.9	4.6
Total dietary fiber	20.1	63.4	7.0	19.4	6.7	16.2
Crude ash	7.4	23.3	6.4	17.7	6.4	15.5
Potassium	0.8	2.5	0.7	1.9	0.7	1.7
Sodium	0.5	1.6	0.5	1.4	0.4	1.0
EPA + DHA	0.33	1.0	0.37	1.0	0.41	1.0
Metabolizable energy *	3171 kcal/kg		3613 kcal/kg		4126 kcal/kg	
Ingredients	Vegetable fibers, maize ^†^, animal fats ^†^, rice ^†^, dehydrated poultry proteins ^†^, wheat ^†^, maize gluten ^†^, wheat gluten ^†^, dehydrated pork protein ^†^, hydrolyzed animal proteins ^†^, minerals, beet pulp, soya oil ^†^, psyllium husks and seeds, yeast products, fish oil ^†^, fructo-oligosaccharides (0.48%), algal oil *Schizochytrium* sp. (source of DHA) ^†^, yeasts (source of mannan-oligosaccharides) (0.19%), marigold meal.		Dehydrated poultry protein ^†^, rice ^†^, rice flour ^†^, maize ^†^, hydrolyzed animal proteins ^†^, animal fats ^†^, beet pulp, egg powder ^†^, vegetable fibers, minerals, soya oil ^†^, yeast products ^†^, fish oil ^†^, psyllium husks and seeds, fructo-oligosaccharides (0.48%), mono- and diglycerides of palmitic and stearic acids esterified with citric acid, algal oil *Schizochytrium* sp. (source of DHA) ^†^, yeasts (source of mannan-oligosaccharides) (0.19%), marigold meal.		Rice ^†^, dehydrated poultry protein ^†^, maize ^†^, animal fats ^†^, hydrolyzed animal proteins ^†^, egg powder ^†^, yeast products ^†^, beet pulp, soya oil ^†^, vegetable fibers, minerals, fish oil ^†^, psyllium husks and seeds, fructo-oligosaccharides, algal oil *Schizochytrium* sp. (source of DHA) ^†^, yeasts (source of mannan-oligosaccharides), marigold meal.	

* Calculated with the predictive equation NRC 2006a (based on crude fiber) [23]. ^†^ Highly digestible ingredients. DHA, docosahexaenoic acid; EPA, eicosapentaenoic acid; GI, gastrointestinal.

**Table 2:** Baseline demographics and clinical characteristics.

	Italian Study			Australian Study	Pooled Australian + Italian Data
	GI High-Fiber Diet (*n* = 21)	GI Moderate-Calorie Diet (*n* = 22)	GI Diet (*n* = 18)	GI High-Fiber Diet (*n* = 14)	GI High-Fiber Diet (*n* = 35)
Male, *n* (%)	13 (61.9)	6 (27.3)	7 (38.9)	6 (42.9)	19 (54.3)
Entire	9 (69.2)	4 (66.7)	5 (71.4)	1 (16.7)	10 (52.6)
Neutered	4 (30.8)	2 (33.3)	2 (28.6)	5 (83.3)	9 (47.4)
Female, *n* (%)	8 (38.1)	16 (72.7)	11 (61.1)	8 (57.1)	16 (45.7)
Entire	2 (25.0)	4 (25.0)	8 (72.7)	0	2 (12.5)
Neutered	6 (75.0)	12 (75.0)	3 (27.3)	8 (100)	14 (87.5)
Age, median (range) years	7.3 (1.0–12.5)	4.2 (1.7–14.0)	4.8 (1.2–11.0)	2.9 (1.3–12.5)	6.0 (1.0–12.5)
Body weight, median (range) kg	29.5 (9.0–53.0)	14.9 (2.4–45.0)	20.2 (12.5–37.1)	17.7 (4.2–38.6)	22.0 (4.2–54.0)
BCS, median (range) *	5.0 (2.0–9.0)	6.0 (1.0–8.0)	4.0 (1.0–5.0)	4.8 (2.0–7.0)	5.0 (2.0–9.0)
mCCECAI score, median (range)	8.0 (5.0–14.0)	6.0 (5.0–15.0)	6.0 (5.0–16.0)	6.0 (3.0–13.0)	7.0 (3.0–14.0)
Disease severity, *n* (%)					
Mild: mCCECAI score 3–5	5 (23.8)	5 (22.7)	3 (16.7)	3 (21.4)	8 (22.9)
Moderate: mCCECAI score 6–8	9 (42.9)	13 (59.1)	10 (55.6)	8 (57.1)	17 (48.6)
Severe: mCCECAI score ≥ 9	7 (33.3)	4 (18.2)	5 (27.8)	3 (21.4)	10 (28.6)
Discomfort caused by GI signs, median (range) score ^†^	8.0 (2.0–9.0)	7.0 (2.0–8.0)	6.0 (2.0–10.0)	5.5 (1.0–10.0)	7.0 (1–10)
Quality of life score, median (range) ^‡^	7.0 (3.0–10.0)	8.0 (6.0–10.0)	8.0 (3.0–10.0)	5.0 (1.0–10.0)	7 (1–10)

* Baseline BCS was not reported for four dogs in the Australian study. ^†^ Discomfort was rated by owners on a scale from 0 (no discomfort) to 10 (extreme discomfort). ^‡^ Quality of life was rated by owners on a scale from 0 (poor quality) to 10 (excellent quality). BCS, body condition score; mCCECAI, modified Canine Chronic Enteropathy Clinical Activity Index; CI, confidence interval, GI; gastrointestinal.

**Table 3:** mCCECAI scores: least squares means and percentage changes from baseline.

Diet	Day	Animals, *n*	Vet-Reported Clinical Score LSM (95% CI)	Percentage Change from Baseline
Italian data only				
GI high-fiber diet	0	21	8.1 (6.9–9.3)	NA
	30	21	2.1 (1.5–2.8)	−73.5 (*p* < 0.0001)
	60	21	1.1 (0.6–1.7)	−85.9 (*p* < 0.0001)
GI moderate-calorie diet	0	22	7.3 (6.1–8.4)	NA
	30	22	1.5 (0.8–2.2)	−79.4 (*p* < 0.0001)
	60	22	1.0 (0.5–1.5)	−86.3 (*p* < 0.0001)
GI diet	0	18	7.2 (5.9–8.5)	NA
	30	18	1.4 (0.7–2.2)	−79.8 (*p* < 0.0001)
	60	17	1.1 (0.5–1.6)	−85.0 (*p* < 0.0001)
Pooled Italian + Australian data				
GI high-fiber diet	0	35	7.4 (6.5–8.4)	NA
	30	33	2.3 (1.7–2.8)	−69.6 (*p* < 0.0001)
	60	31	2.1 (1.6–2.8)	−70.8 (*p* < 0.0001)
Australian data only				
GI high-fiber diet	0	14	6.8 (5.6–8.0)	NA
	30	12	2.4 (1.1–3.7)	−64.7 (*p* < 0.0001)
	60	10	3.2 (1.8–4.5)	−53.4 (*p* = 0.0001)

mCCECAI, modified Canine Chronic Enteropathy Clinical Activity Index; CI, confidence interval; GI, gastrointestinal; LSM, least squares mean; NA, not applicable.

**Table 4:** Owner-reported clinical scores: least squares means and percentage changes from baseline (Italian study only).

Diet	Day	Animals, *n*	Owner-Reported Clinical Score LSM (95% CI)	Percentage Change from Baseline
GI high-fiber diet	Day 0	21	7.0 (6.0–8.0)	NA
	Day 15	21	2.5 (1.5–3.5)	−64.6 (*p* < 0.0001)
	Day 30	21	2.0 (1.3–2.6)	−72.1 (*p* < 0.0001)
	Day 60	21	0.7 (0.2–1.2)	−89.8 (*p* < 0.0001)
GI moderate-calorie diet	Day 0	22	6.5 (5.5–7.5)	NA
	Day 15	22	2.2 (1.2–3.2)	−65.5 (*p* < 0.0001)
	Day 30	22	1.2 (0.5–1.8)	−81.7 (*p* < 0.0001)
	Day 60	22	0.5 (−0.01–0.9)	−93.0 (*p* < 0.0001)
GI diet	Day 0	18	5.8 (4.7–6.9)	NA
	Day 15	18	2.5 (1.4–3.6)	−56.7 (*p* < 0.0001)
	Day 30	18	1.2 (0.4–1.9)	−79.8 (*p* < 0.0001)
	Day 60	17	0.6 (0.1–1.1)	−90.2 (*p* < 0.0001)

CI, confidence interval; GI, gastrointestinal; LSM, least squares mean; NA, not applicable.

**Table 5:** Clinical response to diet at Day 30 (Italian study only).

	GI Diet Group			ORs of a Having a Complete Response: Contrasts Between GI Diet Groups					
	High-Fiber	Moderate-Calorie	GI	High-Fiber vs. Moderate-Calorie		High-Fiber vs. GI		Moderate-Calorie vs. GI	
	n/N (%)	n/N (%)	n/N (%)	%-Point Difference	OR (95% CI)	%-Point Difference	OR (95% CI)	%-Point Difference	OR (95% CI)
Complete response	8/21 (38.1)	14/22 (63.6)	11/18 (61.1)	−25.5	0.4 (0.1–1.2)	−23.0	0.4 (0.1–1.4)	2.5	1.1 (0.3–4.0)
Partial response	13/21 (61.9)	8/22 (36.4)	7/18 (38.9)	25.5	-	23.0	-	−2.5	-

CI, confidence interval; GI, gastrointestinal; OR, odds ratio.

**Figure A1:**
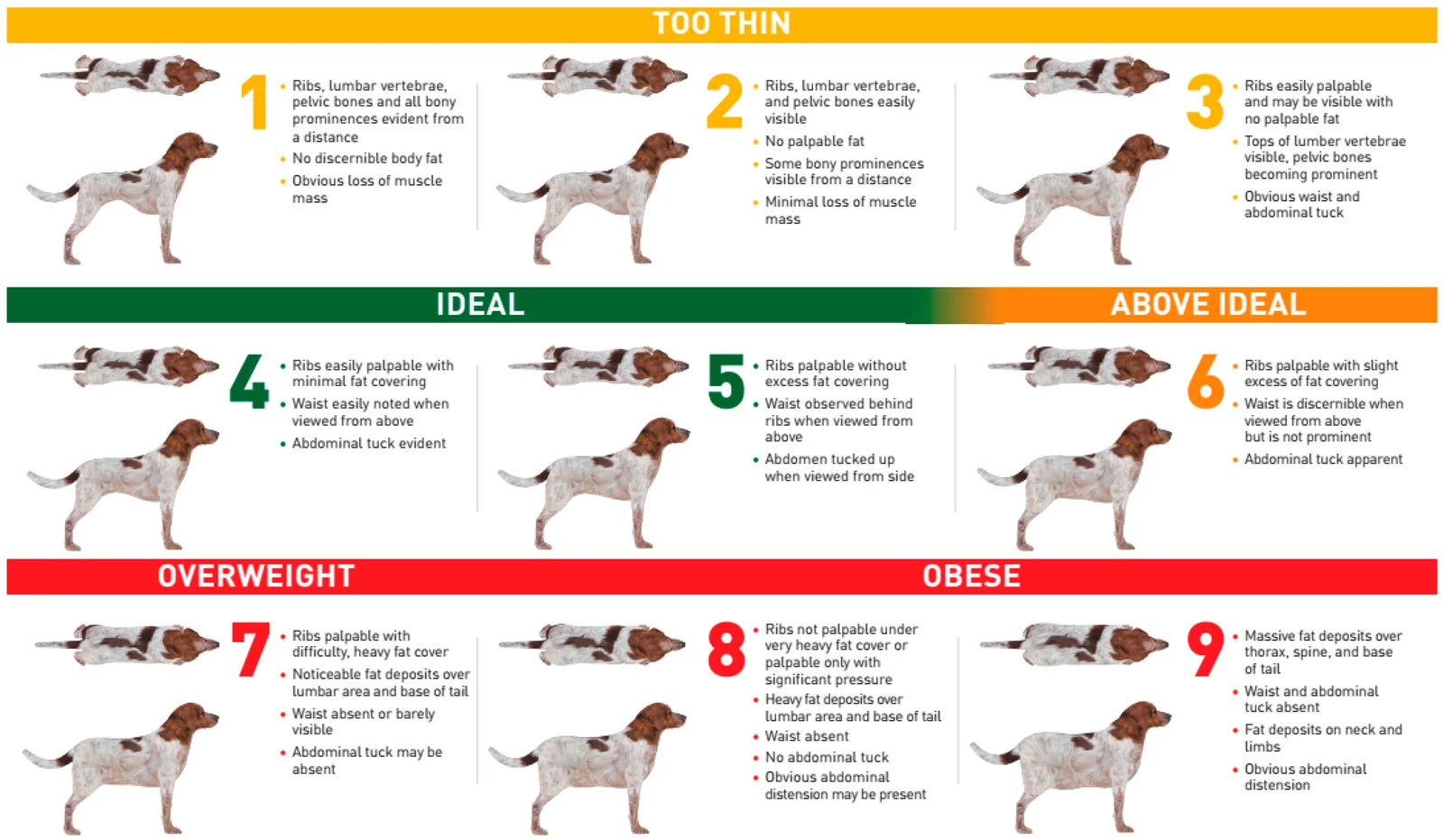
Body condition scores on a scale from 1 = too thin to 9 = obese [22].

**Figure A2:**
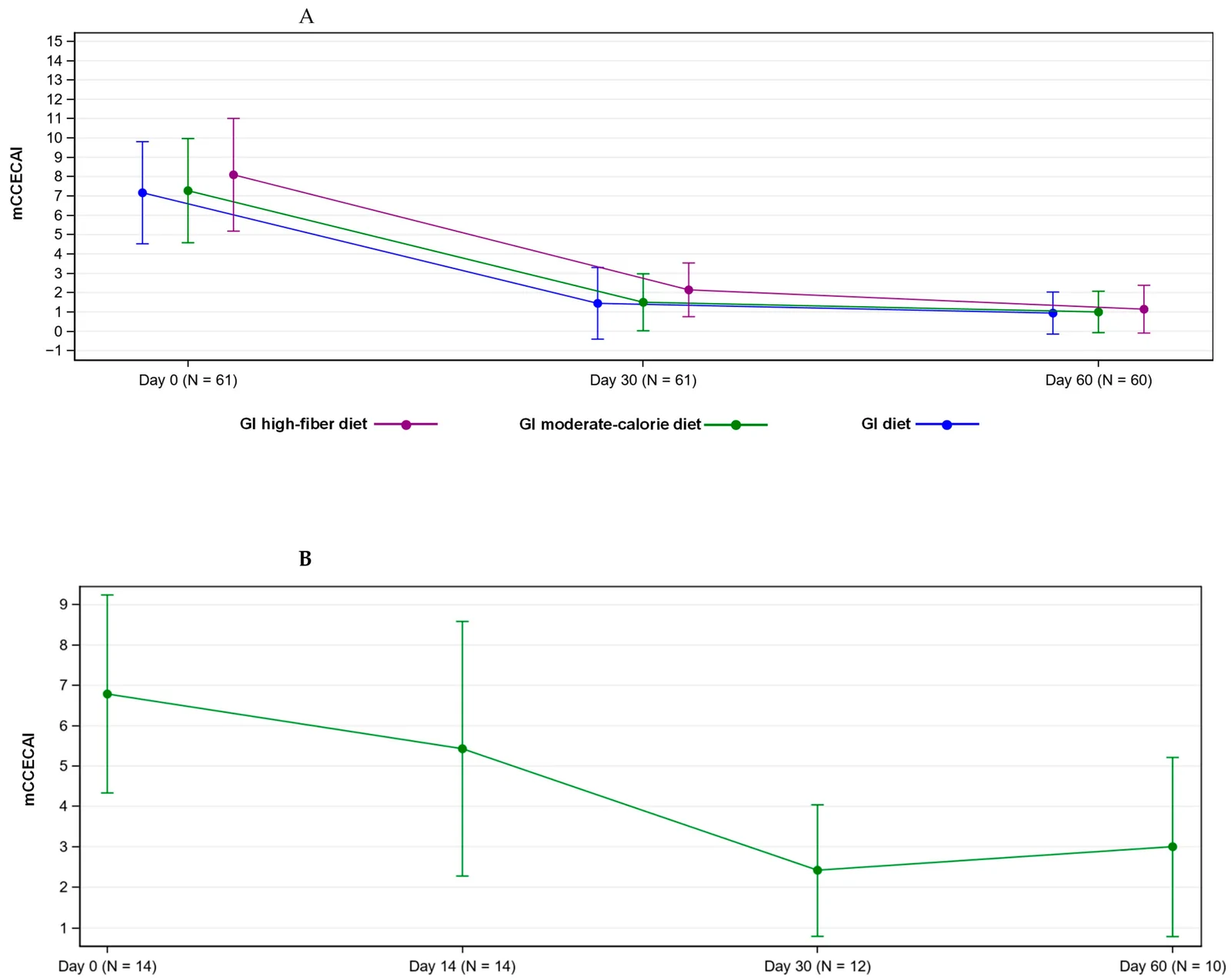
Descriptive statistics for mCCECAI scores over time. Data are arithmetic mean and standard deviation for the three diets in Italian study (**A**) and the GI high-fiber diet in the Australian study (**B**). mCCECAI, modified Canine Chronic Enteropathy Clinical Activity Index.

**Figure A3:**
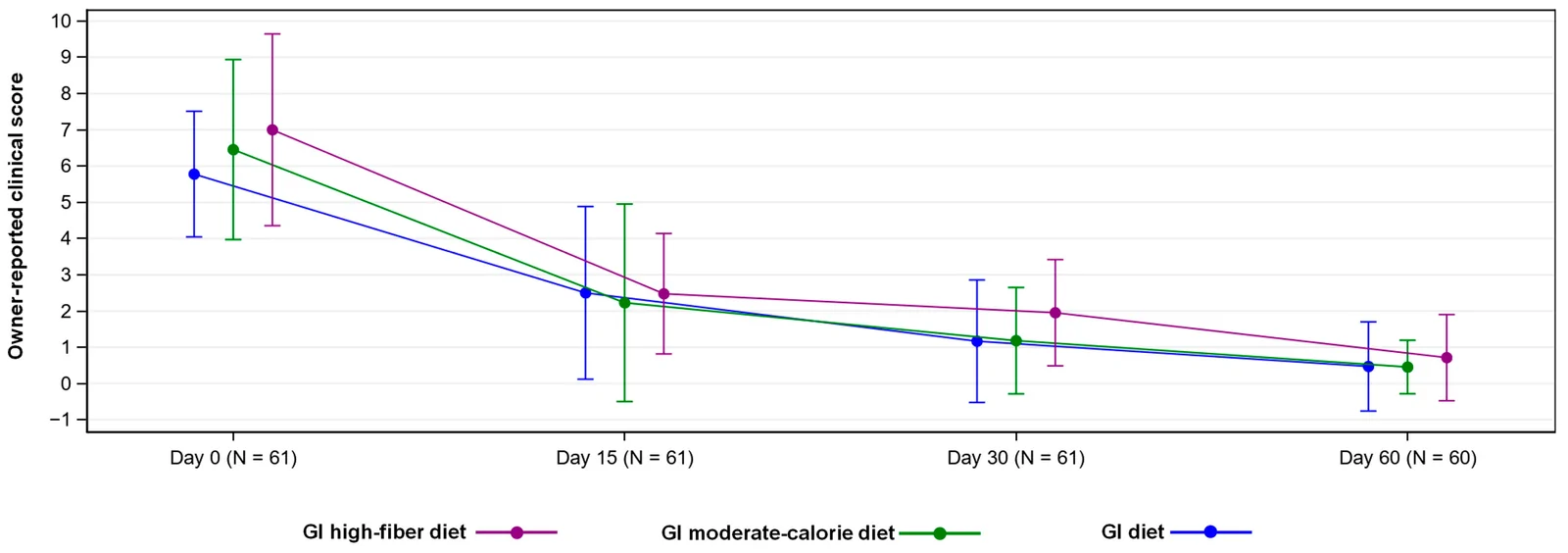
Descriptive statistics for owner-reported clinical scores over time. Data are arithmetic mean and standard deviation for the Italian study only.

## Data Availability

Due to the sensitive nature of the data, anonymized data are available from the corresponding author upon reasonable request.

## Abbreviations

The following abbreviations are used in this manuscript:

BCS	Body Condition Score
CCECAI	Canine Chronic Enteropathy Clinical Activity Index
mCCECAI	Modified Canine Chronic Enteropathy Clinical Activity Index
CIE	Chronic inflammatory enteropathy
CI	Confidence interval
FOS	Fructo-oligosaccharide
GI	GI
LSM	Least squares mean
MOS	Mannan-oligosaccharide
OR	Odds ratio
SCFA	Short-chain fatty acid

## Appendix A

**Table A1:** Vet-reported clinical score (mCCECAI).

Item	Scoring	Scoring Guidance
Attitude/activity	0 = normal	NA
	1 = slightly decreased	
	2 = moderately decreased	
	3 = severely decreased	
Appetite	0 = normal	NA
	1 = slightly decreased	
	2 = moderately decreased	
	3 = severely decreased	
Vomiting	0 = normal	No vomiting
	1 = mild	≤1 vomiting/week
	2 = moderate	2–3 vomiting/week
	3 = severe	>3 vomiting/week
Stool consistency	0 = normal	
	1 = slightly soft feces	
	2 = very soft feces	
	3 = watery diarrhea	
Stool frequency	0 = normal	<2 per day
	1 = slightly increased	2–3 per day or 1–3 per day with fecal blood, mucus or both
	2 = moderately increased	4–5 per day
	3 = severely increased	>5 per day
Weight loss	0 = none	Body weight stable or increased
	1 = mild	<5% weight loss
	2 = moderate	5–10% weight loss
	3 = severe	>10% weight loss
Ascites and peripheral edema	0 = none	No fluid accumulation detected
	1 = mild	Limited ascites (only detectable on imaging) or slight swelling (palpable but not obvious)
	2 = moderate	Palpable ascites (positive fluid wave sign) or clear swelling (visible at a distance)
	3 = severe	Marked abdominal distention, presence of pleural effusion, and severe swelling
Pruritus	0 = no pruritus	
	1 = occasional episodes of itching	
	2 = regular episodes of itching but stops when dog is asleep	
	3 = dog regularly wakes up because of itching	

The mCCECAI score is derived from a previously reported CCECAI score [6]. mCCECAI: modified Canine Chronic Enteropathy Clinical Activity Index.

**Table A2:** Owner-reported clinical score.

Clinical Sign	Scoring for Each Clinical Sign
Attitude	0 = normal or none
Appetite	1 = mild or slight modification
Vomiting	2 = moderate modification
Stool consistency	3 = severe modification
Stool frequency	

**Table A3:** Body weight: least squares means and percentage changes from baseline.

Diet	Day	Animals, *n*	Body Weight, LSM (95% CI) kg	Percentage Change from Day 0 (*p*-Value)
Italian data only				
GI high-fiber diet	Day 0	21	28.3 (23.7–33.0)	NA
	Day 30	21	28.4 (23.8–33.0)	0.2 (0.633)
	Day 60	21	28.3 (23.7–32.8)	−0.2 (0.772)
GI moderate-calorie diet	Day 0	22	16.7 (12.2–21.2)	NA
	Day 30	22	16.7 (12.3–21.2)	0.1 (0.943)
	Day 60	22	16.6 (12.2–21.1)	−0.5 (0.671)
GI diet	Day 0	18	21.0 (16.0–26.0)	NA
	Day 30	18	20.9 (16.0–25.9)	−0.2 (0.812)
	Day 60	17	20.8 (15.9–25.8)	−0.6 (0.593)
Italian and Australian data combined				
GI high-fiber diet	Day 0	35	22.9 (18.7–27.0)	–
	Day 30	33	22.7 (18.7–26.7)	−0.7 (0.283)
	Day 60	31	22.7 (18.7–26.7)	−0.8 (0.214)
Australian data only				
GI high-fiber diet	Day 0	14	27.4 (10.8–24.0)	NA
	Day 30	12	17.0 (10.7–23.3)	−2.2 (0.156)
	Day 60	10	17.1 (10.7–23.4)	−1.9 (0.237)

CI, confidence interval; GI, gastrointestinal; LSM, least squares mean; NA, not applicable.

**Table A4:** Body condition score: least squares means and percentage changes from baseline.

Diet	Day	Animals, *n*	BCS, LSM (95% CI)	Percentage Change from Day 0 (*p*-Value)
Italian data only				
GI high-fiber diet	Day 0	21	5.0 (4.3–5.8)	NA
	Day 30	21	5.1 (4.5–5.8)	1.9 (0.329)
	Day 60	21	5.1 (4.5–5.7)	0.9 (0.720)
GI moderate-calorie diet	Day 0	22	5.9 (5.2–6.6)	NA
	Day 30	22	5.8 (5.2–6.5)	−1.5 (0.340)
	Day 60	22	5.8 (5.1–6.4)	−2.3 (0.295)
GI diet	Day 0	18	3.7 (2.9–4.4)	NA
	Day 30	18	3.8 (3.1–4.5)	3.0 (0.292)
	Day 60	17	4.0 (3.3–4.7)	8.0 (0.046)
Italian and Australian data combined				
GI high-fiber diet	Day 0	31	5.0 (4.3–5.6)	NA
	Day 30	31	5.3 (4.8–5.9)	7.3 (0.011)
	Day 60	30	5.3 (4.7–5.8)	6.0 (0.151)
Australian data only				
GI high-fiber diet	Day 0	10	4.9 (3.9–5.9)	NA
	Day 30	10	5.5 (5.0–6.0)	12.3 (0.123)
	Day 60	9	5.4 (4.8–5.9)	9.3 (0.376)

BCS, body condition score; CI, confidence interval; GI, gastrointestinal; LSM, least squares mean; NA, not applicable.

**Table A5:** Frequency of food consumption scores and odds of a higher score in one versus another diet (Italian study only).

	GI Diet Group			Contrasts Between GI Diet Groups					
Day and Consumption Score	High-Fiber	Moderate-Calorie	GI	High-Fiber vs. Moderate-Calorie		High Fiber vs. GI		Moderate-Calorie vs. GI	
	n/N (%)	n/N (%)	n/N (%)	%-Point Difference	OR (95% CI)	%-Point Difference	OR (95% CI)	%-Point Difference	OR (95% CI)
Day 15					0.2 (0.06–1.3)		0.6 (0.1–3.0)		2.1 (0.5–8.5)
7	0	1/22 (4.5)	0	NA		NA		NA	
8	3/21 (14.3)	7/22 (31.8)	4/18 (22.2)	−17.5		−7.9		9.6	
9	18/21 (85.7)	14/20 (63.6)	14/18 (77.8)	22.1		−7.9		−14.1	
Day 30		-			0.9 (0.2–4.0)		0.5 (0.1–2.1)		0.5 (0.1–2.1)
1	0	0	1/18 (5.6)	NA		NA		NA	
7	2/21 (9.5)	0	1/18 (5.6)	NA		4.0		NA	
8	2/21 (9.5)	5/22 (22.7)	4/18 (22.2)	−13.2		−12.7		0.5	
9	17/21 (81.0)	17/22 (77.3)	12/18 (66.7)	3.7		−14.3		10.6	
Day 60					1.1 (0.3–4.0)		1.9 (0.4–7.9)		1.7 (0.4–7.0)
7	3/21 (14.3)	2/22 (9.1)	0	5.2		NA		NA	
8	4/21 (19.0)	5/22 (22.7)	4/17 (23.5)	−3.7		−4.5		−0.8	
9	14/21 (66.7)	15/22 (68.2)	13/17 (76.5)	−1.5		−9.8		−8.3	

At each timepoint, owners were asked: on average, how much of the product did your dog eat compared to the dose prescribed? Owners selected a consumption score of 1 to 9, where 9 indicated that the dog had consumed the full maximum daily allowance. The table only shows scores that were given for at least one dog at the timepoint. At each timepoint, the OR for Diet A vs. Diet B is the odds that Diet A will have a higher consumption score than Diet B. CI, confidence interval; GI, gastrointestinal; OR, odds ratio; NA, not applicable.

**Table A6:** Owner-rated scores of dogs’ discomfort caused by GI problems. Data are for dogs fed the GI high-fiber diet in the Australian study only.

Day	Animals, *n*	Discomfort Score, LSM (95% CI)	Percentage Change from Day 0 (*p*-Value)
Day 0	14	5.2 (3.5–6.89)	–
Day 14	14	2.5 (1.5–3.5)	−52.1 (0.017)
Day 30	11	1.78 (0.89–2.7)	−66.1 (0.001)
Day 60	10	1.2 (0.5–1.9)	−77.4 (<0.0001)

Discomfort was rated on a scale from 0 (no discomfort) to 10 (extreme discomfort). CI, confidence interval; GI, gastrointestinal; LSM, least squares mean.

**Table A7:** Owner-rated scores of dogs’ quality of life. Data are for dogs fed the GI high-fiber diet in the Australian study only.

Day	Animals, *n*	Discomfort Score, LSM (95% CI)	Percentage Change from Day 0 (*p*-Value)
Day 0	14	5.6 (3.5–7.6)	NA
Day 14	14	4.3 (2.22–6.3)	−23.1 (0.366)
Day 30	11	4.01 (1.8–6.3)	−27.1 (0.299)
Day 60	10	3.5 (1.1–5.8)	−37.8 (0.115)

Quality of life was rated on a scale from 0 (poor quality) to 10 (excellent quality). CI, confidence interval; GI, gastrointestinal; LSM, least squares mean; NA, not applicable.
